# Hypersensitivity to BK_Ca_ channel opening in persistent post-traumatic headache

**DOI:** 10.1186/s10194-024-01808-0

**Published:** 2024-06-18

**Authors:** Haidar M. Al-Khazali, Rune H. Christensen, David W. Dodick, Basit Ali Chaudhry, Anna G. Melchior, Rami Burstein, Håkan Ashina

**Affiliations:** 1 Harvard Medical School, Boston, MA USA; 2 Department of Neurology, BIDMC Comprehensive Headache Center, Beth Israel Deaconess Medical Center, Boston, MA USA; 3 Department of Neurology, Danish Headache Center, Copenhagen University Hospital – Rigshospitalet, Copenhagen, Denmark; 4 Department of Clinical Medicine, Faculty of Health and Medical Sciences, University of Copenhagen, Copenhagen, Denmark; 5 Translational Research Center, Copenhagen University Hospital – Rigshospitalet, Copenhagen, Denmark; 6 Department of Neurology, Mayo Clinic, Scottsdale, AZ USA; 7 Department of Anesthesia, Critical Care and Pain Medicine, Beth Israel Deaconess Medical Center, Harvard Medical School, Center for Life Science, 3 Blackfan Circle, Boston, MA 02215 USA

**Keywords:** BK_Ca_ channels, Head pain, Pathogenesis, Drug targets, Head trauma

## Abstract

**Background:**

Large conductance  calcium-activated potassium (BK_Ca_) channels have been implicated in the neurobiological underpinnings of migraine. Considering the clinical similarities between migraine and persistent post-traumatic headache (PPTH), we aimed to examine whether MaxiPost (a BK_Ca_ channel opener) could induce migraine-like headache in persons with PPTH.

**Methods:**

This is a randomized double-blind, placebo-controlled, two-way crossover study from September 2023 to December 2023. Eligible participants were adults with PPTH after mild traumatic brain injury who reported having no personal history of migraine. The randomized participants received a single dose of either MaxiPost (0.05 mg/min) or placebo (isotonic saline) that was infused intravenously over 20 minutes. The two experiment sessions were scheduled at least one week apart to avoid potential carryover effects. The primary endpoint was the induction of migraine-like headache after MaxiPost as compared to placebo within 12 hours of drug administration. The secondary endpoint was the area under the curve (AUC) values for headache intensity scores between MaxiPost and placebo over the same 12-hour observation period.

**Results:**

Twenty-one adult participants (comprising 14 females and 7 males) with PPTH were enrolled and completed both experiment sessions. The proportion of participants who developed migraine-like headache was 11 (52%) of 21 participants after MaxiPost infusion, in contrast to four (19%) participants following placebo (*P* = .02). Furthermore, the median headache intensity scores, represented by AUC values, were higher following MaxiPost than after placebo (*P* < .001).

**Conclusions:**

Our results indicate that BK_Ca_ channel opening can elicit migraine-like headache in persons with PPTH. Thus, pharmacologic blockade of BK_Ca_ channels might present a novel avenue for drug discovery. Additional investigations are nonetheless needed to confirm these insights and explore the therapeutic prospects of BK_Ca_ channel blockers in managing PPTH.

**ClinicalTrials.gov Identifier:**

NCT05378074.

**Supplementary Information:**

The online version contains supplementary material available at 10.1186/s10194-024-01808-0.

## Introduction

Persistent post-traumatic headache (PPTH) is a debilitating neurological disorder, which often results from mild traumatic brain injury (mTBI) [1, 2]. The appropriate treatment for those affected is unclear, with no evidence-based standard of care [3, 4]. Thus, identifying novel drug targets for PPTH remains an important unmet need. In this context, large conductance calcium-activated potassium (BK_Ca_) channels might constitute a promising target for drug development.

Emerging evidence has shed light on the role of BK_Ca_ channels in the neurobiological underpinnings of head pain, [5] particularly in migraine [6]. This observation warrants attention due to the striking clinical parallels between migraine and PPTH [7, 8]. Recent insights also suggest a shared pathophysiological basis for both disorders [9]. For instance, experimental studies have identified the involvement of calcitonin gene-related peptide (CGRP) and pituitary adenylate cyclase-activating polypeptide (PACAP) in the pathogenesis of both migraine and PPTH [10–13].

The role of CGRP and PACAP is intriguing, as both of them bind to their respective G protein-coupled receptors on the vascular smooth muscle cells (VSMCs) within the meningeal arteries, [14–16] facilitating the opening of BK_Ca_ channels [17]. This process results in vasodilation and increased levels of extracellular potassium ions which, in turn, might discharge perivascular meningeal nociceptors [18, 19]. Yet, the potential contribution of BK_Ca_ channel opening to the development of migraine-like headache in persons with PPTH remains an enigma.

To bridge this knowledge gap, we carried out a randomized, double-blind, placebo-controlled, two-way crossover study. Our aim was to investigate whether administration of MaxiPost (a BK_Ca_ channel opener) could induce migraine-like headache in persons with PPTH who reported having no personal history of migraine.

## Methods

The study protocol was approved by the Regional Health Research Ethics Committee of the Capital Region of Denmark (H-21048424). The participants were recruited from a tertiary referral hospital and provided written informed consent prior to enrollment. The study was conducted in accordance with the standards of the Declaration of Helsinki. All authors have approved the manuscript and attest to the accuracy and completeness of the data. The protocol is available in the Supplemental Appendix.

### Participants

The study included persons between the ages of 18 and 65 years who had a diagnosis of PPTH resulting from mTBI, as outlined in the International Classification of Headache Disorders, 3^rd^ edition (ICHD-3) [20]. Eligible participants also had to experience an average of at least 4 headache days per month in the three months leading up to their enrollment. Participants were excluded if they had any pre-mTBI history of a headache disorder, except for infrequent episodic tension-type headache. In addition to this, persons with multiple mTBIs or recent alterations in their dosage of preventive headache medication were also excluded. The complete list of inclusion and exclusion criteria is available in the Supplemental Appendix.

### Design

The study design has been published in detail elsewhere and is briefly described here [13, 21]. A randomized, double-blind, placebo-controlled, two-way crossover design was used. The preparation of MaxiPost and placebo was carried out by independent pharmacy staff. The administered dose of MaxiPost (0.05 mg/mL) corresponded to the one used in prior experimental studies involving persons with migraine and healthy volunteers [5, 6]. The independent pharmacy staff were also responsible for allocation concealment and randomization in block sizes of 4 for the first 20 participants enrolled. The last participant was then separately randomized and allocated to receive either MaxiPost or placebo. This was done to ensure that the principles of randomization were upheld and to avoid any potential selection bias.

The randomized participants were assigned to receive a single dose of either MaxiPost (0.05 mg/min) or placebo (isotonic saline) that was infused intravenously over 20 minutes. Both interventions were delivered using a time- and volume-controlled infusion pump. To avoid potential carryover effects, the two experiment sessions were scheduled at least one week apart. The experiment session was postponed if the participants had taken acute headache medication within 48 hours prior to their scheduled infusion. The same applied for participants who presented with a migraine-like headache or reported a headache intensity exceeding 3 on an 11-point numeric rating scale at the time of their scheduled infusion.

### Procedures

Prior to the first experiment session, participants underwent a semi-structured interview to collect their demographic and clinical data. A neurologic examination was then performed, and the participants were informed about the possible onset or exacerbation of head pain after MaxiPost infusion. However, details concerning the onset, duration, or features of the head pain were not disclosed.

The order of events during the 2 experiment sessions is shown in the Supplemental Appendix. Trained personnel established intravenous access via peripheral cannulation of the antecubital fossa with the participant in a supine position. A paper diary was then introduced as an evaluation instrument for the participant to record symptoms, medication use, and adverse events. The first entry was made at infusion start and then every 10 minutes for the first hour. Thereafter, participants were discharged and asked to record entries once hourly from 2 to 12 hours post-infusion start.

### Endpoints

The primary endpoint was the induction of migraine-like headache after MaxiPost as compared to placebo within 12 hours of drug administration. The participants were classified as having experienced a migraine-like headache if they met the criteria outlined in Table 1. The secondary endpoint was the area under the curve (AUC) values for headache intensity scores between MaxiPost and placebo over the same 12-hour observation period. Headache intensity was rated using an 11-point numeric rating scale, ranging from 0 (lowest score) to 10 (highest score). Additional exploratory endpoints were the AUC values for the percentage change in mean arterial blood pressure and heart rate after MaxiPost as compared to placebo within 1 hour of drug administration.

**Table 1:** Criteria for migraine-like headache

The following criteria are used for experimentally induced migraine-like headache:
Migraine-like headache must fulfill at least two of the following four characteristics:
• Unilateral location
• Pulsating quality
• Moderate or severe pain intensity
• Aggravation by or causing avoidance of routine physical activity (e.g., walking or climbing stairs)
During headache, at least one of the following must be fulfilled:
• Nausea and/or vomiting
• Photophobia and phonophobia, or
• Headache mimicking the usual headache exacerbation with migraine-like features

### Statistical analysis

A sample size of 21 participants was determined to provide 80% power at a one-sided significance level of .05. This assumed that 50% experience migraine-like headache only following MaxiPost and 10% do so only after placebo. The sample size calculations were performed using McNemar’s test.

Descriptive statistics were applied to present demographic and clinical characteristics of the study population. For continuous variables, we reported means with standard deviations (SD) or medians with interquartile ranges, as appropriate. The data distribution was evaluated for normality using both the Shapiro-Wilk test and visual inspection. For categorical data, we reported absolute numbers and percentages.

Analysis of the primary endpoint was performed using McNemar’s test. Outcome data was included from all randomized participants in accordance with the intention-to-treat principle. For the secondary endpoint, baseline-correction was performed to minimize the impact of variations in headache intensities at infusion start between the two experiment sessions.^10,12,20^ The trapezium method was then used to determine the AUC for each time point of data entry during the 12-hour observation period. Following this, we used the Wilcoxon signed-rank test to compare the AUC values between MaxiPost and placebo. Moreover, paired t-tests were used to analyze the baseline-corrected results of the two exploratory endpoints, and a binomial regression model was applied to test for potential carryover effects.

## Results

**Fig. 1:**
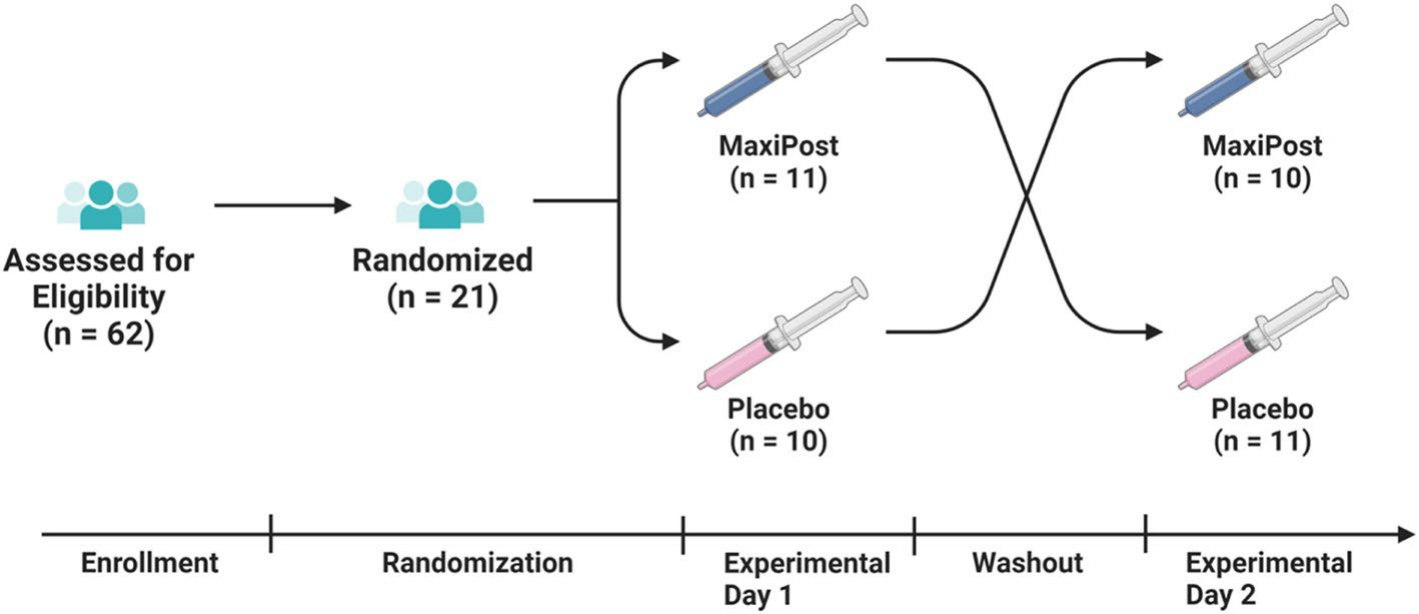
Study Flow Diagram. n, number.

A total of 62 persons were screened and 21 underwent randomization (Fig. 1). The participants’ mean age was 44.0 (SD, 11.9) years, with most being females (*n* = 14) and reporting a migraine-like phenotype (*n* = 20). The median time elapsed since their mTBI was 8.0 (IQR, 5.5 to 10.5) years, and most participants (*n* = 18) experienced headache on ≥15 days per month. In addition, four (19%) participants reported a positive family history of migraine, and three (14%) had ongoing use of preventive headache medication. The demographic and clinical characteristics of the study population is presented in Table 2.

**Table 2:** Demographic and clinical characteristics of the study population

Characteristics	**Persistent Post-Traumatic Headache, *n* = 21**
Age, mean ± SD, years	44.0 ± 11.9
Body Mass Index, mean ± SD, kg/m^2^	26.6 ± 4.4
Family History of Migraine without Aura, n (%)	4 (19)
Family History of Migraine with Aura, n (%)	0 (0)
Time since TBI, median (IQR), years	8.0 (5.5 to 10.5)
Migraine-Like Phenotype, n (%)	20 (95.2)
Tension-Type Headache-Like Phenotype, n (%)	1 (4.8)
Monthly Headache Days, mean ± SD	26.6 (8.3)
Current Use of Acute Headache Medication, n (%)	19 (90)
Current Use of Preventive Headache Medication, n (%)	3 (14.3)

*SD* Standard deviation, *IQR* Interquartile range

### Headache responses

**Fig. 2:**
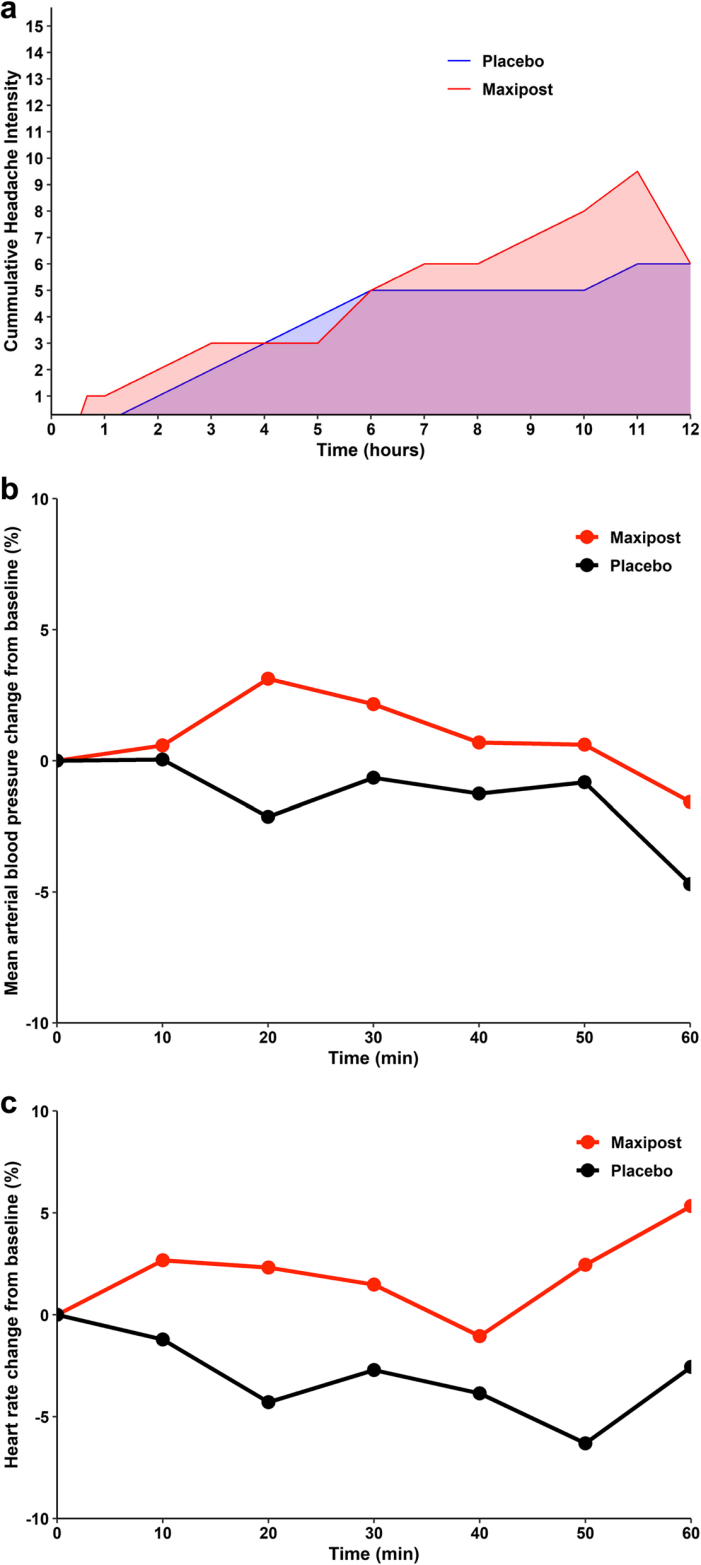
**a** Baseline-Corrected Median Cumulative Headache Intensity Scores after MaxiPost and Placebo. Baseline-corrected median cumulative headache intensity scores after MaxiPost and placebo infusion during the 12-hour observation window. The red line denotes median cumulative headache intensity scores after MaxiPost infusion, whilst the blue line denotes median cumulative headache intensity scores after placebo. **b** Baseline-Corrected Mean Arterial Blood Pressures after MaxiPost and Placebo. Baseline-corrected mean arterial blood pressure values after MaxiPost and placebo infusion during the 60-min in-hospital period. The red line denotes mean arterial blood pressure after MaxiPost, whilst the black line denotes mean arterial blood pressure after placebo. **c** Baseline-Corrected Mean Heart Rates after MaxiPost and Placebo. Baseline-corrected mean heart rate values after MaxiPost and placebo infusion during the 60-min in-hospital period. The red line denotes mean heart rates after MaxiPost, whilst the black line denotes mean heart rates after placebo

Eleven (52%) of 21 participants developed migraine-like headache over the 12-hour observation period post-MaxiPost infusion. This contrasted with four (19%) participants after placebo (*P* = .02). Of interest, seven participants recorded experiencing migraine-like headache only after MaxiPost infusion. Four participants developed migraine-like headache after both MaxiPost and placebo, whilst no participants did so exclusively after placebo. Among the 11 participants with migraine-like headache after MaxiPost infusion, the median onset time was 120 (IQR, 40 to 300) minutes. A more detailed overview is shown in the Supplemental Appendix. Furthermore, the median headache intensity scores, represented by AUC values, were higher after MaxiPost infusion relative to placebo (*P* < .001 after baseline-correction; Fig. 2a).

### Peak headache characteristics

Among the 21 participants, the peak headache intensity had a median value of 4 (IQR, 3 to 6) after MaxiPost, in contrast to a median of 1 (IQR, 1 to 4) after placebo. Of interest, the peak headache was observed at a median time of 120 (IQR, 28 to 375) minutes post-MaxiPost infusion. This headache was primarily of bilateral location (*n* = 14), pressing quality (*n* = 16), and moderate or severe pain intensity (*n* = 14). Eleven participants also experienced pain worsening after routine physical activity. Common accompanying symptoms were photophobia (*n* = 14), phonophobia (*n* = 10), and nausea or vomiting (*n* = 5).

### Reported adverse events and rescue medication use

The most common adverse events were facial flushing, palpitations, and warm sensations; all of which were more prevalent following MaxiPost than after placebo (*P* < .05 for each event). No serious adverse events occurred. Moreover, four (19%) of 21 participants resorted to rescue medication to alleviate their headache following MaxiPost infusion, whereas only one participant needed to do so after receiving placebo.

### Hemodynamic responses

There were no significant differences between the percentage change in mean arterial blood pressure, represented by AUC values, following MaxiPost infusion, compared to placebo (*P* = .2426; Fig. 2b). Similar to this, no significant differences were identified in the AUC values for percentage change in mean heart rate after MaxiPost infusion in comparison to placebo (*P* = .1787; Fig. 2c).

## Discussion

Our randomized, placebo-controlled study discovered that MaxiPost, a BK_Ca_ channel opener, can induce migraine-like headache in persons with PPTH who reported no pre-mTBI history of migraine. Importantly, the participants reported that their spontaneous migraine-like headache closely mirrored the one induced by MaxiPost. This observation bolsters our assertion that BK_Ca_ channel opening can generate cephalic pain, particularly migraine-like headache, among persons with PPTH. Thus, our findings pave the way for the development of BK_Ca_ channel blockers as a novel approach to address unmet treatment needs in managing PPTH.

### BK_Ca_ Channel Involvement in Headache Disorders

The contribution of BK_Ca_ channels to the pathogenesis of headache disorders has garnered increasing attention in recent years [17]. Randomized, placebo-controlled studies have found that intravenous infusion of MaxiPost can induce migraine attacks in persons with migraine [6]. In contrast, healthy individuals only experience non-migraine-like headache following MaxiPost infusion [5]. Moreover, experimental studies have shown that intravenous infusion of MaxiPost results in decreased blood flow velocity within the middle cerebral artery [5, 6]. This observation holds true for both persons with migraine and healthy individuals [5, 6]. Thus, BK_Ca_ channel openers appear to dilate intracranial arteries, a physiologic effect that is consistent across all known triggers of migraine-like headache [12, 22, 23]. Although it might seem intuitive that MaxiPost can induce migraine-like headache in people with PPTH, given that it induces migraine attacks in people with migraine, this assumption is not necessarily universally applicable across molecular headache triggers. For instance, despite CGRP being a well-known migraine inducer [10, 24, 25] it does not trigger migraine attacks in people with familial hemiplegic migraine [26, 27]. Likewise, a K_ATP_ channel opener, levcromakalim, induced migraine attacks in all participants with migraine without aura, [28] but the incidence of migraine-like headache in people with PPTH and cluster attacks in those with cluster headache, is clearly lower [21, 29]. This differential response is also evident in our findings, where 52% of participants with PPTH experienced migraine-like headache post-MaxiPost infusion, as opposed to a 95% induction rate in people with migraine [6]. Given the identical dosage and infusion durations used across both studies, it is plausible that BK_Ca_ channel opening plays a less prominent role in the pathogenesis of PPTH, compared with migraine. Furthermore, the molecular and cellular mechanisms underlying PPTH could differ from those of migraine, which might account for the varied responses to the same molecular headache trigger. Taken together, there is evidently variability in sensitivities and expression of different headache phenotypes in response to molecular headache triggers. This underscores the intricate interplay of molecules in headache pathogenesis, which goes beyond specific headache phenotypes. It is also important to recognize the challenge of a priori predicting individual responses to molecular headache triggers across different headache disorders. Therefore, randomized, placebo-controlled studies are essential for an in-depth evaluation of these responses. Taken together, the involvement of BK_Ca_ channels seem to cover a plethora of physiological processes, including regulation of intracranial vasculature, neuronal excitability, and pain transmission, among others.

### Proposed sites and mechanisms of action

**Fig. 3:**
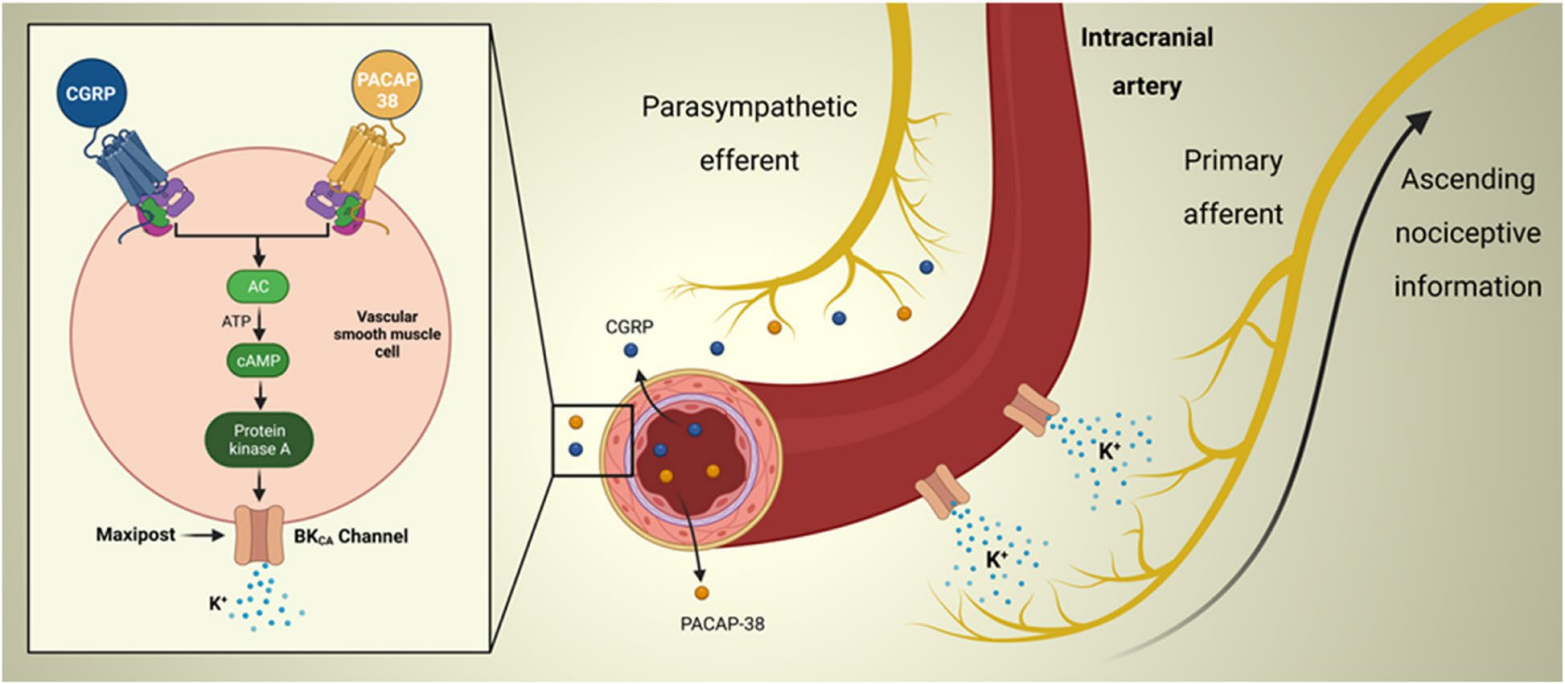
Possible Mechanisms and Sites of Action of Migraine-Like Headache induced by BK_Ca_ Channel Opening in Persons with PPTH. The figure outlines a proposed mechanism and site of action though which opening of BK_Ca_ channels contributes to the development of migraine-like headache in persons with PPTH. In this suggested model, the signaling molecules CGRP and PACAP-38 bind to their respective G protein-coupled receptors present on the vascular smooth muscle cells of intracranial arteries. This initiates the activation of cAMP-dependent signaling pathways, which then results in opening of BK_Ca_ channels. The subsequent release of potassium ions and accompanying vasodilation leads to activation and sensitization of perivascular meningeal nociceptors, a process facilitated by both chemical and mechanical stimulation. Modified from Al-Khazali et al., 2023 [21]. CGRP = calcitonin gene-related peptide; PACAP-38 = pituitary adenylate cyclase-activating polypeptide-38; AC = adenylate cyclase; ATP = adenosine triphosphate; cAMP = cyclic adenosine monophosphate; BKCa channels = large conductance calcium-activated potassium channels

The site(s) and mechanism(s) through which the activation of BK_Ca_ channels culminate in migraine-like headache continue to be areas of active exploration. One plausible site of action is the meninges and its vasculature (Fig. 3). Prior research indicates that MaxiPost promotes dilation of intracranial arteries in both persons with migraine and healthy individuals [5, 6]. This vasodilatory effect is corroborated by preclinical data, demonstrating a causal link between the BK_Ca_ channel opening and dilation [30]. A proposed mechanism involves the efflux of potassium ions from VSMCs, leading to vasodilation [17].^17^ The rationale is that increased extracellular levels of potassium, in part, due to BK_Ca_ channel opening offer electrochemical stimuli, while the concurrent vasodilation provides mechanical stimuli. Together, these stimuli might have the potential to activate perivascular nociceptors. The culmination of this cascade is hypothesized to result in migraine-like headache [18, 19]. This proposed sequence of events aligns well with emergent evidence indicating that administration of CGRP and PACAP, both potent vasodilators of meningeal arteries, [22, 31] can induce migraine-like headache in persons with PPTH [11, 13]. It is worth noting that this vasodilatory response is mediated, in part, through the activation of BK_Ca_ channels [17]. Taken together, future rodent experiments should ascertain whether BK_Ca_ channel opening within the meningeal vasculature can indeed activate perivascular nociceptors.

An alternate, albeit less probable, site of action might be the primary afferent fibers whose cell bodies are located in the trigeminal ganglion and upper cervical ganglia. These fibers are responsible for the nociceptive innervation of the meninges, including its vasculature [32]. Opening of the BK_Ca_ channels causes the membrane potential to become more negative, causing a hyperpolarization of the afferent fibers. The implication is then that administration of a BK_Ca_ channel opener, such as MaxiPost, will exert anti-nociceptive effects, which is incongruent with our findings. However, a possible explanation might be that BK_Ca_ channel opening on the primary afferent fibers can activate hyperpolarization-activated cyclic nucleotide-gated (HCN) channels. This, in turn, can result in depolarization and possible nociceptor activation [33–35]. Nonetheless, this hypothesis remains speculative and less convincing in light of preclinical evidence showing that iontophoretic application of a BK_Ca_ channel opener inhibits primary afferent fibers. In addition, preclinical data suggest that MaxiPost’s direct effect on nociceptive neurons within the CNS is unlikely, as bath application of a BK_Ca_ channel opener inhibited nociceptive responses in 2^nd^ order trigeminal neurons, whereas blocking these channels facilitated nociceptive responses [36].

Furthermore, the human provocation model has shown that CGRP infusion not only triggers migraine headache but also its accompanying symptoms [10, 24, 25]. Conversely, antibodies not only reduce headache and migraine days but also reduce the accompanying symptoms of migraine [37–39]. The mechanism is not yet known. Preclinical studies have shown that CGRP's downstream effect includes phosphorylation and opening of BK_Ca_ channels, which is why BK_Ca_ opening with Maxipost is also assumed to have a similar effect by activating these accompanying symptoms [40]. Al-Kharagoli et al. also found a significant difference in the incidence of accompanying symptoms (i.e, photophobia, phonophobia, and nausea) with Maxipost compared to placebo among migraine patients [6]. Therefore, it could be hypothesized that blocking BK_Ca_ channels would likely also inhibit the induction of these symptoms.

### Therapeutic implications and future directions

Our results lend support to the pivotal role of BK_Ca_ channels in the pathogenesis of PPTH. If the opening of BK_Ca_ channels can trigger head pain, it is plausible that blocking these channels might alleviate the pain. The development of BK_Ca_ channel blockers might thus represent a novel and targeted approach to treating PPTH. However, while our findings are promising, they represent only the first step towards novel drug discovery. BK_Ca_ channels are known for their ubiquitous expression across various tissues, including those implicated in the genesis of head pain, such as the intracranial arteries and trigeminal ganglion [40, 41]. The development of effective and safe BK_Ca_ channel blockers will therefore require extensive research. In this context, it will be important to explore the exact sites and mechanisms of BK_Ca_ channel action within the neurobiological underpinning of PPTH.

An issue that merits special emphasis is when to initiate pharmacologic treatment with a BK_Ca_ channel blocker in persons with PPTH. Here, one might consider that CGRP’s downstream action in VSMCs involve BK_Ca_ channel opening, and inhibition of CGRP signaling can attenuate cutaneous allodynia in concussed rodents. This effects appears, however, to decrease over time, suggesting a time-sensitive window for optimal pharmacologic treatment post-concussion. This time-bound phenomenon might be intimately tied to diffuse noxious inhibitory controls (DNIC), a physiological regulator of descending pain modulation [42]. A disruption in DNIC function, as seen in concussed rodents, might reduce the brain’s ability to modulate ascending pain signals, thereby leading to the development of persistent and chronic headache. Here, it warrants mention that the loss of DNIC function can be prevented in concussed rodents after inhibition of CGRP signaling [43]. The question is then whether a BK_Ca_ channel blocker could replicate similar outcomes in these rodent models. Should this be the case, it suggests the importance of early treatment with a BK_Ca_ channel blocker after mTBI for the management of PPTH.

### Limitations

This study has some limitations that warrant mention. First, the in-hospital observation period extended only to one hour following the infusion due to logistical considerations. Thus, we cannot exclude the potential influence of various environmental factors, such as dietary intake or stress levels, on the data collected throughout the remaining 11 hours of the observation period. Second, the use of rescue medication presents another limitation, albeit a minimal one given its infrequent usage among our participants to alleviate headache symptoms. Third, the use of a placebo in our two-way crossover design directly addresses the concern regarding the potential overlap between spontaneous and MaxiPost-induced migraine-like headache in our participants. Using placebo, we can effectively differentiate between migraine-like headache that occurs spontaneously and those induced by MaxiPost. This design allows each participant to serve as their own control, thereby mitigating the influence of natural headache fluctuations. Hence, we can more accurately attribute differences in headache responses to the effects of MaxiPost, rather than natural variations in headache occurrence. Fourth, ongoing use of preventive headache medication might have mitigated the headache response after MaxiPost infusion. This highlights the need for further research to elucidate the potential impact of these variables on the initiation and evolution of migraine-like headache in PPTH. Fifth, in addition to Maxipost's primary role in opening BK_Ca_ channels, it also modulates GABAA receptors and neuronal Kv7 channels [44]. These interactions are expected to mitigate neuronal hyperexcitability, suggesting potential therapeutic applications in pain management. Yet, human experimental studies have clearly demonstrated that MaxiPost is a potent molecular headache trigger [5, 6]. It remains uncertain whether modulation of GABAA receptors and neuronal Kv7 channels contribute to or counteract MaxiPost’s ability to trigger headache. This adds a layer of complexity in interpreting our findings, and further research is required to clarify the roles of GABAA receptors and neuronal Kv7 channels in headache pathogenesis. Lastly, it cannot be excluded that some participants might be predisposed to migraine, which was then elicited in response to head trauma. Their migraine-like headache might, therefore, represent a genuine migraine attack. According to the ICHD-3 [20], people with migraine can experience an exacerbation of their typical migraine attacks after head trauma. It is, however, important to note that our participants reported no personal history of migraine and developed acute PTH within seven days of the head trauma. Further research is warranted to determine whether head trauma can indeed ‘activate’ a latent predisposition to migraine, a topic that has garnered attention in recent literature [45, 46].

## Conclusions

Our findings reveal that intravenous infusion of a BK_Ca_ channel opener can elicit migraine-like headache in persons with PPTH. This sparks therapeutic promise in developing BK_Ca_ channel blockers to manage PPTH. Yet, more research is warranted to unravel the role of BK_Ca_ channel in the disease mechanisms underlying PPTH.

### Supplementary Information

Supplementary Material 1. 

## Data Availability

The data that support the results of this study are available from the corresponding author upon reasonable request.

## Abbreviations

PPTH: Persistent post-traumatic headache
mTBI: Mild traumatic brain injury
BK_Ca_: Large conductance calcium-activated potassium
CGRP: Calcitonin gene-related peptide
PACAP-38: Pituitary adenylate cyclase-activating polypeptide
VSMCs: Vascular smooth muscle cells
ICHD-3: International Classification of Headache Disorders, 3rd edition
SD: Standard deviations
IQR: Interquartile ranges
AUC: Area under the curve
HCN: Hyperpolarization-activated cyclic nucleotide-gated
DNIC: Diffuse noxious inhibitory controls
