# Supplementary material for: Hypersensitivity to BKCa channel opening in persistent post-traumatic headache
Source: J Headache Pain. 2024 Jun 18;25(1):102. doi: 10.1186/s10194-024-01808-0 (PMC11186171; doi:10.1186/s10194-024-01808-0)
Supplement: Supplementary file 1 — Supplementary Material 1. [file 10194_2024_1808_MOESM1_ESM.docx]

**Supplemental Appendix.**

**Supplemental Table 1. Inclusion Criteria**

| Inclusion Criteria | Data Source |
| --- | --- |
| Age 18 to 65 years of age upon entry into screening | Legal identification document |
| History of persistent headache attributed to mild traumatic injury to the head for ≥ 12 months and in accordance with the International Classification of Headache Disorders, 3^rd^ Edition (ICHD-3) | Medical record and/or subject self-report as assessed by site investigator during the semi-structured interview |
| ≥ 4 monthly headache days on average across the 3 months prior to screening | Subject self-report as assessed by site investigator during the semi-structured interview |
| Provision of informed consent prior to initiation of any study-specific activities/procedures. | Informed consent form |

**Supplemental Table 2. Exclusion Criteria.**

| Exclusion Criteria | Data Source |
| --- | --- |
| > 1 mild traumatic injury to the head | Medical record and/or subject self-report as assessed by site investigator |
| History of any primary or secondary headache disorder prior to mild traumatic injury to the head (except for infrequent episodic tension-type headache) | Medical record and/or subject self-report as assessed by site investigator during the semi-structured interview |
| History of moderate or severe injury to the head | Medical record and/or subject self-report as assessed by site investigator during the semi-structured interview |
| History of whiplash injury | Medical record and/or subject self-report as assessed by site investigator during the semi-structured interview |
| History of craniotomy | Medical record and/or subject self-report as assessed by site investigator during the semi-structured interview |
| History or evidence of any other clinically significant disorder, condition or disease (except for those outlined above) than, in the opinion of the site investigator, would pose a risk to subject safety or interfere with study evaluation, procedures or completion | Medical record and/or subject self-report as assessed by site investigator during the semi-structured interview |
| The subject is at risk of self-harm or harm to others as evidenced by past suicidal behavior | Medical record and/or subject self-report as assessed by site investigator |
| Female subjects of childbearing potential with a positive pregnancy test during any study visit | Human chorionic gonadotropin (hCG) test (urine) |
| Cardiovascular disease of any kind, including cerebrovascular diseases | Medical record and/or subject self-report as assessed by site investigator during the semi-structured interview |
| Hypertension (systolic blood pressure of ≥150 mmHg and/or diastolic blood pressure of ≥100 mmHg) prior to the start of infusion on the experimental day | Blood pressure measurement |
| Hypotension (systolic blood pressure of ≤90 mmHg and/or diastolic blood pressure of ≤50 mmHg) | Blood pressure measurement |
| Initiation, discontinuation, or change of dosing of prophylactic medications within 2 months prior to study inclusion | Medical record and/or subject self-report as assessed by site investigator during the semi-structured interview |
| Intake of acute medications (e.g. analgesics, triptans) within 48 hours of infusion start | Subject self-report as assessed by site investigator during the semi-structured interview |
| Baseline headache intensity of >3 on an 11-point numeric rating scale (0 being no headache, 10 being the worst imaginable headache) | Subject self-report as assessed by site investigator during the semi-structured interview |
| Baseline headache with migraine-like features or self-reported baseline headache that mimics the subjects’ usual headache with migraine-like features | Subject self-report as assessed by site investigator during the semi-structured interview |

**Supplemental Table 3. Characteristics of Migraine-Like Headache after Maxipost and Placebo.**

| Participant No./Sex | Headache Phenotype | Type of Intervention | Time to Peak Headache (min) | Peak Headache Characteristics^a^ | Mimics Usual Migraine-Like Headache^b^ | Migraine-Like Headache^c^ (Time to Onset) | Worsening of Associated Symptoms^d^ |
| --- | --- | --- | --- | --- | --- | --- | --- |
| **1/Female** | Migraine-Like | Maxipost  Placebo  Usual | 20 min  120 min | Bilat/5/Pres/+  Bilat/4/Pres/-  Bilat/8/Throb/- | No  No | No  No | NA  NA |
| **2/Male** | Migraine-Like | Maxipost  Placebo  Usual | 420 min  540 | Bilat/5/Pres/-  Bilat/6/Pres/-  Bilat/8/Throb/+ | No  No | No  No | NA  NA |
| **3/Male** | Migraine-Like | Maxipost  Placebo  Usual | 120 min  300 min | Unilat/4/Pres/-  Unilat/5/Pres/-  Unilat/6/Pres/+ | No  No | No  No | NA  NA |
| **4/Male** | Migraine-Like | Maxipost  Placebo  Usual | 0 min  360 min | Bilat/3/Pres/-  Bilat/4/Pres/-  Bilat/4/Throb/+ | No No | No  No | NA  NA |
| **5/Female** | Migraine-Like | Maxipost  Placebo  Usual | 0 min  0 min | Unilat/3/Pres/-  Bilat/3/Pres/-  Unilat/5/Throb/+ | No  No | No  No | NA  NA |
| **6/Female** | Migraine-Like | Maxipost  Placebo  Usual | 40 min  180 min | Bilat/7/Pres/+  Bilat/4/Pres/+  Bilat/9/Throb/+ | Yes  No | Yes (20 min)  No | +/+/-  NA |
| **7/Male** | Migraine-Like | Maxipost  Placebo  Usual | 240 min  40 min | Bilat/4/Pres/+  Bilat/7/Pres/-  Bilat/6/Comb/+ | Yes  No | Yes (40 min)  No | -/+/+  NA |
| **8/Female** | Migraine-Like | Maxipost  Placebo  Usual | 300 min  40 min | Unilat/3/Throb/+  Unilat/3/Throb/+  Bilat/7/Pres/+ | No  No | Yes (300 min)  Yes (20 min) | -/+/+  -/+/+ |
| **9/Female** | Migraine-Like | Maxipost  Placebo  Usual | 420 min  10 min | Bilat/7/Pres/+  Bilat/2/Pres/-  Bilat/7/Comb/+ | Yes  No | Yes (300 min)  No | +/+/-  NA |
| **10/Female** | Migraine-Like | Maxipost  Placebo  Usual | 720 min  420 min | Bilat/9/Throb/+  Bilat/3/Throb/-  Bilat/7/Throb/+ | Yes  No | Yes (420 min)  No | +/+/+  NA |
| **11/Female** | Migraine-Like | Maxipost  Placebo  Usual | 720 min  180 min | Bilat/6/Pres/+  Bilat/5/Throb/+  Bilat/4/Throb/+ | No  No | Yes (30 min)  Yes (30 min) | +/+/+  +/+/+ |
| **12/Male** | Migraine-Like | Maxipost  Placebo  Usual | NA  120 min | NA  Bilat/2/Pres/-  Unilat/8/Pres/- | No  No | No  No | NA  NA |
| **13/Female** | Migraine-Like | Maxipost  Placebo  Usual | 120 min  180 min | Bilat/6/Pres/+  Bilat/6/Pres/+  Bilat/5/Pres/+ | No  No | Yes (120 min)  Yes (120 min) | +/+/+  -/+/+ |
| **14/Female** | Migraine-Like | Maxipost  Placebo  Usual | 480 min  0 min | Bilat/4/Pres/-  Bilat/3/Pres/-  Bilat/6/Pres/+ | No  No | No  No | NA  NA |
| **15/Female** | Migraine-Like | Maxipost  Placebo  Usual | 300 min  20 min | Bilat/6/Throb/-  Bilat/4/Pres/-  Bilat/7/Throb/+ | Yes  No | Yes (360 min)  No | -/+/+  NA |
| **16/Female** | Migraine-Like | Maxipost  Placebo  Usual | 30 min  0 min | Bilat/2/Pres/-  Bilat/1/Pres/-  Unilat/10/Throb/+ | No  No | No  No | NA  NA |
| **17/Female** | Migraine-Like | Maxipost  Placebo  Usual | 40 min  0 min | Bilat/5/Pres/+  Bilat/3/Pres/+  Bilat/4/Throb/+ | Yes  No | Yes (30 min)  No | -/+/+  NA |
| **18/Male** | Migraine-Like | Maxipost  Placebo  Usual | 120 min  20 min | Unilat/4/Pres/+  Bilat/4/Pres/+  Unilat/8/Pres/+ | Yes  No | Yes (120 min)  No | -/+/+  NA |
| **19/Female** | TTH-Like | Maxipost  Placebo  Usual | 10 min  20 min | Bilat/1/Pres/-  Bilat/3/Pres/-  Bilat/5/Pres/- | No  No | No  No | NA  NA |
| **20/Female** | Migraine-Like | Maxipost  Placebo  Usual | 360 min  10 min | Bilat/4/Pres/+  Bilat/1/Pres/-  Bilat/4/Comb/+ | Yes  No | Yes (360)  Yes (20) | +/+/+  +/+/+ |
| **21/Male** | Migraine-Like | Maxipost  Placebo  Usual | 0 min  0 min | Bilat/3/Throb/-  Bilat/3/Throb/-  Bilat/9/Throb/+ | No  No | No  No | NA  NA |

No, number; TTH, tension-type headache; Bilat, bilateral; Throb, throbbing; Unilat, unilateral; Pres, pressing: NA, not applicable; Comb, combined throbbing and pressing quality of headache.

a Localization (unilateral, bilateral) / pain intensity (11-point numeric rating scale, with 0 indicating no headache and 10 indicating the worst headache imaginable) / quality of headache (throbbing, pressing, combined throbbing and pressing) / aggravation of headache by routine physical activity (plus denotes presence, minus denotes absence).

b The participant is asked to determine whether the headache following Maxipost or placebo infusion resembles his/her, if applicable, usual migraine-like headache.

c Migraine-like headache is defined by the criteria outlined in Table 1.

Associated symptoms are nausea, photophobia, and phonophobia. Worsening is defined as an increase in the severity of these symptoms at the onset of migraine-like headache compared with baseline (i.e., time of infusion start), as rated on a 4-point Likert scale (0 = none, 1 = mild, 2 = moderate, 3 = severe).
